# From peacetime to war: a path analysis of the factors that predict performance among police and military commanders in collaborative crisis response

**DOI:** 10.3389/fpsyg.2023.1238760

**Published:** 2023-12-22

**Authors:** Jostein Mattingsdal, Jan Aandal, Bjørn Helge Johnsen, Roar Espevik

**Affiliations:** ^1^Royal Norwegian Naval Academy, Norwegian Defense University College, Laksevåg, Norway; ^2^Emergency Response Team Selection and Training Department, Oslo Police, Oslo, Norway; ^3^Center for Crisis Psychology, Faculty of Psychology, University of Bergen, Bergen, Norway; ^4^Leadership and Command and Control Division, Swedish Defense University, Stockholm, Sweden

**Keywords:** hybrid warfare, decision-making, collaborative crisis response, organizational performance, persistence, collective self-efficacy, police-military interoperability

## Abstract

**Purpose:**

This study aimed to examine the applicability of Bandura’s social cognitive theory in predicting organizational performance in dynamic and ambiguous hybrid warfare contexts. Specifically, the study investigated the influence of dyad composition, past performance in peacetime, collective self-efficacy, and persistence on wartime performance among high-ranking police and military commanders.

**Study design/methodology/approach:**

One hundred and thirty-eight participants, consisting of police and military commanders, took part in a simulation exercise that escalated from peace to war. The participants were assigned to three types of dyads (*N* = 69); all-police (*n* = 20), all-military (*n* = 27), and mixed police-military (*n* = 22). The study utilized path analysis to examine the direct and indirect effects of the variables on wartime performance.

**Results:**

The model developed in this study accounted for 54% of the variance in wartime performance (*R*^2^ = 0.54). Path analysis showed direct effects of persistence (β = −0.33) and peacetime performance (β = 0.45) on actual performance in wartime. Direct effects also showed how persistence was predicted by dyad composition (β = −0.24) and peacetime performance (β = −0.50). Indirect effects indicated how persistence mediated the effects of peacetime performance (β = 0.17) and dyad composition (β = 0.08) on actual performance in wartime.

**Originality/value:**

This study contributes to the understanding of how social cognitive factors, as described by Bandura’s theory, can predict decision outcomes in collaborative crisis response settings involving police and military commanders. The findings have implications for policy-making and provide recommendations for further research in this area.

## Introduction

A matter of increasing concern is how the ambiguous threats of hybrid warfare,^1^ which are hard to comprehend, let alone counter, challenge the police and military’s ability to collaborate in security crises (Wrange, 2022). As the need for more efficacious crisis response systems is growing, the present article investigates the organizational performance of police and military commanders from the perspective of social cognitive theory (SCT) of Bandura (1999). As used herein, organizational performance refers to the activities commanders engage in while allocating troops to tasks and adapting to change in available resources and situational circumstances (Mintzberg, 1989).

Given SCT’s grounding in the social environment, it can explain organizational functioning at both the individual and group level (Stajkovic and Sergent, 2019, p. 11). SCT provides detailed descriptions of how people’s behaviors and personal circumstances interact with the environment to influence each other through reciprocal determinism (p. 1). In this conceptualization, people are active information seekers that strive for control of matters of perceived importance to achieve desired outcomes. SCT further explains how people make decisions by forming expectations about the anticipated outcomes of actions, by self-reflectively accessing and processing information. Group interactions are thus contingent on the accumulated knowledge and domain-specific skills of those involved (p. 4). Of particular note is how Bandura (1986, p. 394) predicts that the outcomes of these mechanisms may manifest themselves differently in the ways people persist in the face of ambiguity and setbacks. As used herein, persistence refers to the extent to which people mobilize resources and expend efforts in the face of difficulties (Bandura, 1997, p. 76).

In ambiguous conditions, SCT (Wood and Bandura, 1989) defines three features that determine the performance of people working together in the management of organizations: (a) Their selective assessments of the bigger picture and long-term considerations derived from previous experience; (b) Their ability to make the most of their resources while adhering to personal standards and organizational norms; and (c) Their self-regulatory ability to innovate, improvise, and adapt to rapidly changing circumstances. In the current study, we thus believed SCT would be instructive for analyzing the reciprocal relationships between the behaviors and personal factors of high-ranking commanders and the contextual factors of hybrid warfare.

### Background

Many scholars report how self-regulation determines the degree to which people invest in tasks, and how this in turn predicts work-related performance in a number of professional domains (Stajkovic and Luthans, 1998), including rescue work (Prati et al., 2010), nursing (Pham et al., 2019), education (Martin et al., 2009), leadership (McCormick, 2001), business (Nwosu et al., 2022), and teamwork (Staples and Webster, 2007). However, relatively little is known about the processes predicting the organizational performance of governmental officials (Jervis, 2017, p. xvi). Even less is known about the variables linked to organizational performance when several security providers cooperate in the management of hybrid warfare (Caliskan and Liégeois, 2021). On this account, Bandura (1999) posits that the contrasting backgrounds and previous experience of professionals having equal expertise, but distinct standards of adequacy, would play prominent roles when they have to make decisions without fully knowing the extent to which a course of action is justified. As used herein, decision-making refers to the process explaining how group members without conflicts of interest, but with problems of ambiguity, use their domain-specific skills to size up events, form expectancies, determine the reasonable cues and goals, and identify a response and carry it out (Klein, 2017).

To address the organizational performance of police-military command dyads engaged in collaborative crisis response regarding hybrid warfare, we thus evaluate the influence of two fixed social cognitive factors (dyad composition and past performance in peacetime) and two relatively pliable factors (collective self-efficacy and persistence) on organizational performance in wartime. Although Bandura (1997) predicts that these factors are linked to organizational performance, scholars assert that there is still substantial overlap between the concepts, which calls for contextual adaptations (Schunk and DiBenedetto, 2021). For example, research describes how SCT explains group performance in manufacturing (Little and Madigan, 1997), teaching (Goddard et al., 2004), and business (Kim and Shin, 2015). On the other hand, studies have also demonstrated conflicting conclusions concerning the influence of both efficacy beliefs and past performance on actual performance (Heggestad and Kanfer, 2005). However, scholars have thus far not examined the role played by social cognitive factors in explaining organizational performance in war.

To focus our study, the analysis was limited to command dyads at a national headquarters, as this setting not only includes the vicarious management of ongoing events, but also the planning of future actions (Mintzberg, 1989). Dyads are groups of two (Graen and Scandura, 1987) and were used in the current study because dyads can operate under the same principles and theories that explain group processes for groups of three and larger groups (Williams, 2010). Furthermore, our approach follows research encouraging social cognitive investigations in field settings (Schunk and Usher, 2019) and the performance of groups involving people with complementary competencies (Ochoa et al., 2007).

As we focus on the social cognitive factors predicting organizational performance, we believe the potential serious consequences of hybrid attacks on Norway (Diesen, 2018), and the Norwegian police and military’s reputation for acting cooperatively (Spurkland, 2021), make it possible to assume mutual confidence between the current study’s participants. In this regard, we firmly believe that comprehending the specific Western^2^ context of the interactions between Norwegian police and military commanders carries substantial significance for various reasons. These reasons encompass the imperative for individuals occupying positions of authority to adopt strategies in accordance with the prevailing institutional frameworks inherent in open societies. Furthermore, fostering interagency cooperation that strictly adheres to the principles of the rule of law, ensuring transparency, and aligning response measures with the priorities and standards set by the public is of utmost importance (Arneson, 2009). Consequently, by following these democratic principles, it should become possible for police and military commanders to effectively counter hybrid warfare while upholding the ethical expectations of the public and avoiding potential transgressions.

Although the interdependence and mutual aims of police and military commanders in western countries are close enough to consider them a team (Salas et al., 2008), SCT predicts between group decision-making differences as the demands of specific standards and the constraints of ambiguity will assure that different combinations of self-regulatory mechanisms are activated in the face of hybrid warfare. As a result, the properties of choice alternatives will evoke little or much motivation, depending on how a course of action is perceived as useful for achieving desirable outcomes (Bandura, 1999, p. 171).

To this end, we asked the following questions: (1) How does the dyads’ composition, collective self-efficacy, and past performance in peacetime predict their actual performance in wartime? (2) What is the relationship between the dyad’s level of persistence and their performance in wartime? To answer these questions, we used an original data set collected from a simulation involving 138 high-ranking commanders from all branches of the military, and nine out of 12 Norwegian police districts.

### Related work on decision-making in collaborative crisis response

A review of the scholarly discourse reveals the importance of organizational differences, previous experience, and adaptive self-regulation for understanding decision-making in collaborative efforts (Mosier et al., 2018; Usher and Weidner, 2018). Research of policing (Herrington and Colvin, 2015), military operations (Gray, 2015), and interagency operations (Schedler and Kuipers, 2022) supports how personal factors relate to the skillful management of resources across time and space, regardless of who is in charge and who is supporting the accomplishment of tasks (Floyd, 2021). This highlights the paramount significance of understanding the individual decision-makers occupying higher levels of leadership. However, scholars assert that little empirical work is written about the decisions of high-ranking commanders in wartime (Shortland et al., 2019).

On this note, scholars of hybrid warfare discuss how crisis response systems based on highly sectorized decision-making will be challenged when the resources of several sectors are overwhelmed by the number and severity of threats (Weissmann et al., 2021). Nowhere is this more evident than in the 22 July Norway attacks, in which the government failed to mobilize resources that would have been critical if there had been more attacks that day (Gjørv et al., 2012). Similar obstacles to efficient decision-making are exemplified in other hybrid warfare induced security crises, such as the ongoing war in Ukraine involving private military companies (Østensen and Bukkvoll, 2022) and separatist groups supported by Russia (Freedman, 2019). The multifaceted conflict in Syria is another illustrative example, combining conventional and unconventional tactics (Bachmann and Jones, 2021). Additionally, there have been instances of flawed police-military interactions, accompanied by subsequent erratic decision-making regarding hybrid attacks on Israel (Matthews, 2011). These events collectively exemplify an ambiguous decision environment characterized by the involvement of multiple actors, the blending of diverse methods, transboundary implications, information warfare, the necessity for interagency coordination, and the potential for escalation, requiring decision-makers to make timely and prompt decisions.

In the wake of the emergence of hybrid warfare, it has been two recurring debates related to decision-making in collaborative crisis response. The first debate concerns the ambiguous nature of the contemporary security environment (Huddy et al., 2023), wherein hybrid warfare appear as a particularly formidable challenge for decision-makers within the government (Mumford and Carlucci, 2022). This form of warfare introduces substantial risks of excessive crisis escalation among the involved parties (Cullen and Reichborn-Kjennerud, 2017) and is compounded by the heightened accessibility of weapons of mass destruction and the associated challenges they present (Okoro and Oluka, 2019). The second debate concerns how pre-existing decision-making frameworks often yield poor organizational performance in situations that are difficult to predict and anticipate (Marchau et al., 2019). Moreover, these issues are amplified by the growing phenomenon of police militarization (Kraska, 2021) and the blurred boundaries between internal and external security in Western countries (van Vark, 2021), as well as the difficulties of applying traditional rules of engagement to hybrid warfare scenarios (Schmitt et al., 2022).

The ambiguous decision-making environment inherent to hybrid warfare seems especially pertinent in the interface of police and military commanders. Within this domain, their distinct sector-specific responsibilities play a crucial role in governing the support and leadership of collaborative efforts (Thiele, 2021). Both police and military commanders are trained to respond swiftly to crises, through established command and control systems, communication networks, and operational procedures they can activate when needed (Lægreid and Rykkja, 2018). This readiness allows both groups to mobilize resources, deploy personnel, and coordinate response efforts in a timely and coordinated manner. However, as argued by Corbe and Cusumano (2018), their overlapping capabilities also introduce difficulties in coordinating and aligning police-military efforts to effectively address challenges that require collaboration (see Larsson et al. (2023) for an overview of arguments on the social-psychological aspects of civil-military interagency collaboration). Thus, in the current study, we believed that investigating the predictors of performance based on SCT was a crucial first step to test the asserted relationship between interagency collaborations and the resilience of modern societies.

The current study’s rationale is thus inevitably based on the identified deficiencies in the crisis response system of countries such as Norway (Gjørv et al., 2012), Germany (Fleisher, 2014), the United Kingdom (Murphy, 2006), and the United States (Hoffman et al., 2015). Moreover, it takes into account the challenges arising from the distinct and specialized boundaries between the police and military (Auglend, 2016), as well as the increasing vulnerabilities faced by modern societies in general (Larssen, 2021). With this in mind, the primary objective of the current study was to foster enhanced understanding of how police and military commanders can effectively address the challenges posed by hybrid warfare.

### Hypothesis

#### Dyad composition

Research of public administration (Lee and Hung, 2021) and collaborative crisis management (Bynander and Nohrstedt, 2019) asserts that group composition influences performance attainment when public organizations cope with unexpected events under the same resource constraints and mandates that make up their daily routines. Furthermore, research of hybrid warfare suggests that interagency groups, based on members from the police, military, and other governmental and non-governmental security providers, promote operational flexibility that is beneficial for countering hybrid warfare (Tagarev, 2021). Yet researchers studying how professionals make decisions (Schneider and Shanteau, 2003) have rarely taken social cognitive factors into consideration when addressing the influence of group composition on performance in security crises. The effect of unilateral or interagency grouping in higher-level headquarters thus remains unknown. To address this issue, the current study explores the effects of dyad composition (i.e., all-police, all-military, or mixed police-military) on organizational performance in wartime. The following hypothesis was thus put forward:

*Hypothesis 1: Interagency grouping will enhance organizational performance both directly and indirectly by effecting persistence.*

#### Past performance in peacetime

According to Bandura (1986), when commanders manage threats occurring in the ambiguous gray zone between peace and war, they will rely heavily on their previous experience from peacetime to determine which courses of action they prefer. The central question here is the unique contributions of the dyads’ peacetime performance to their actual performance in wartime. As such, positive effects in the past performance/actual performance relationship are reported in fields such as teamwork (Margarida Passos and Caetano, 2005), education (Elias and MacDonald, 2007), sports (Jackson et al., 2020), and project management (Zarghami and Zwikael, 2022). However, Bandura and Jourden (1991) explain that such influences of past performance on actual performance are likely to decrease over time. It is important to note that this effect has only been demonstrated in conditions of low ambiguity (Schmidt and DeShon, 2010). Based on this, we reasoned that since hybrid warfare is characterized by high levels of ambiguity, the dyads’ performance in peacetime should play an important role in determining how efforts are mobilized in war. The following hypothesis was thus put forward:

*Hypothesis 2: High levels of peacetime performance will enhance organizational performance in wartime both directly and indirectly by effecting persistence.*

#### Collective self-efficacy and performance

According to Bandura (1997), collective self-efficacy is a motivational construct explaining how a group’s confidence in its abilities is associated with greater success, so that higher collective self-efficacy leads to better group performance. The relation between collective self-efficacy and group performance is considered robust (Gibson et al., 2000), and strong correlations have been demonstrated in recent meta-analysis (Stajkovic et al., 2009). In the current study, dyads having strong beliefs about their power to produce intended results and effect change through collective action should therefore demonstrate high performance levels. The following hypothesis was thus put forward:

*Hypothesis 3: A strong sense of collective self-efficacy will enhance organizational performance both directly and indirectly by effecting persistence.*

#### Persistence and performance

Social cognitive theory (SCT; Bandura, 1986) describes how people whose standards encourage hard work and persistence should expend more resources in the face of difficulties and consequently be more efficacious than less persistent individuals. Similarly, people with standards that encourage collaboration should be more likely to do so, compared with those who lack previous experience from successful collaborative efforts. Accordingly, commanders who feel competent about overseeing collaborative efforts should persist and mobilize efforts in ways that minimize resource requirements and mitigate risks to friendly actions. Thus, if subordinates report threats, this will substantiate people’s perceptions of progress and motivate the necessary resource allocations (Schunk and DiBenedetto, 2021, p. 155). For such reasons, scholars argue that organizational performance in times of war relies on prudent decisions more than inflexible tenacity (Gray, 2010). In a Norwegian context, the reckless use of force is not only a waste of resources, but also involves risks of excessive escalation. If so, overly persistent efforts and excessive use of force should result in lower performance attainment. Indeed, the relationship between persistence and performance could be argued to be inversely related in such settings.

The ways in which commanders at headquarters need to align tactical actions with national objectives, while managing limited resources (Olsen and Van Creveld, 2010), thus provide an interesting target for testing the predictions of SCT. The extent to which the commanders’ persistence impacts organizational performance in wartime has not yet been investigated, but in accordance with the SCT predictions, we expected that it would play a significant role. The following hypothesis was thus put forward:

*Hypothesis 4: Elevated levels of persistence will reduce the level of organizational performance in wartime.*

## Methods

### Participants

The 138 participants (men/women = 118/20, police/military = 62/76) were balanced by age/seniority and assigned to one of three dyad conditions: (1) Mixed police-military (*n* = 22), all-police (*n* = 20), and all-military (*n* = 27), *N* = 69.

The military participants were drawn from all branches, including the joint headquarters, with ranks ranging from captain to major-general or the equivalent. The police participants were drawn from the police districts and police directorate, with ranks ranging from inspector to assistant chief of police.

All participants had previous leadership experience and an average of 20.19 years of active-duty service, with a standard deviation (SD) of 8.0 years. The average age of the participants was 43.0 years (SD = 7.9).

In the mixed police-military dyads, the average age was 43.8 years (SD = 8.0), while in the all-police dyads it was 44.1 years (SD = 7.9), and in the all-military dyads 41.1 years (SD = 7.7).

### Instruments and variables

The simulation was conducted at a virtual national headquarters using a video conferencing program (i.e., Microsoft Teams) via the secure VPN connections of the Norwegian police and military. The use of video conferencing in both research (Gray et al., 2020) and crisis management (Chandler and Wallace, 2009) is well-documented and is frequently used by government agencies to coordinate activities, due to its security options and cost-effectiveness. We thus reasoned that the simulation’s video conferencing was a close approximation of the actual decision-making environment of commanders at national headquarters.

The researcher took part in the video conference, to initiate and observe the study. Computer software (Microsoft Forms) controlled the sequence of the slides and recorded all the respondents’ decisions. No time limit was assigned to the simulation. The average time to complete was 106.23 min (about 2 h). *Scenario*: To simulate the ambiguous nature of hybrid warfare (Weissmann et al., 2021), the scenario involved tasks that traversed the traditional responsibilities of police and military commanders. These tasks encompassed diverse responsibilities aimed at safeguarding national security and public safety. They included conducting counterintelligence and counterterrorism operations, promptly responding to emergency situations, collecting and analyzing intelligence, ensuring the safety of hostages or individuals affected by crises, safeguarding critical infrastructure, and maintaining border integrity to prevent illicit activities.

Geographically, the scenario’s threats targeted Norway’s critical infrastructure in an arc from Svalbard in the north to Skagerrak in the south, constituting a major security crisis. To ensure that the simulation included the strategic dilemmas of new and emerging security threats, the current scenario was based on NATO’s Occasus exercise module (Derksen, 2018). Events were described by realistic intelligence products, such as assessments of the adversaries’ capabilities, friendly forces information, geospatial data, and civil considerations.

**Figure 1:**
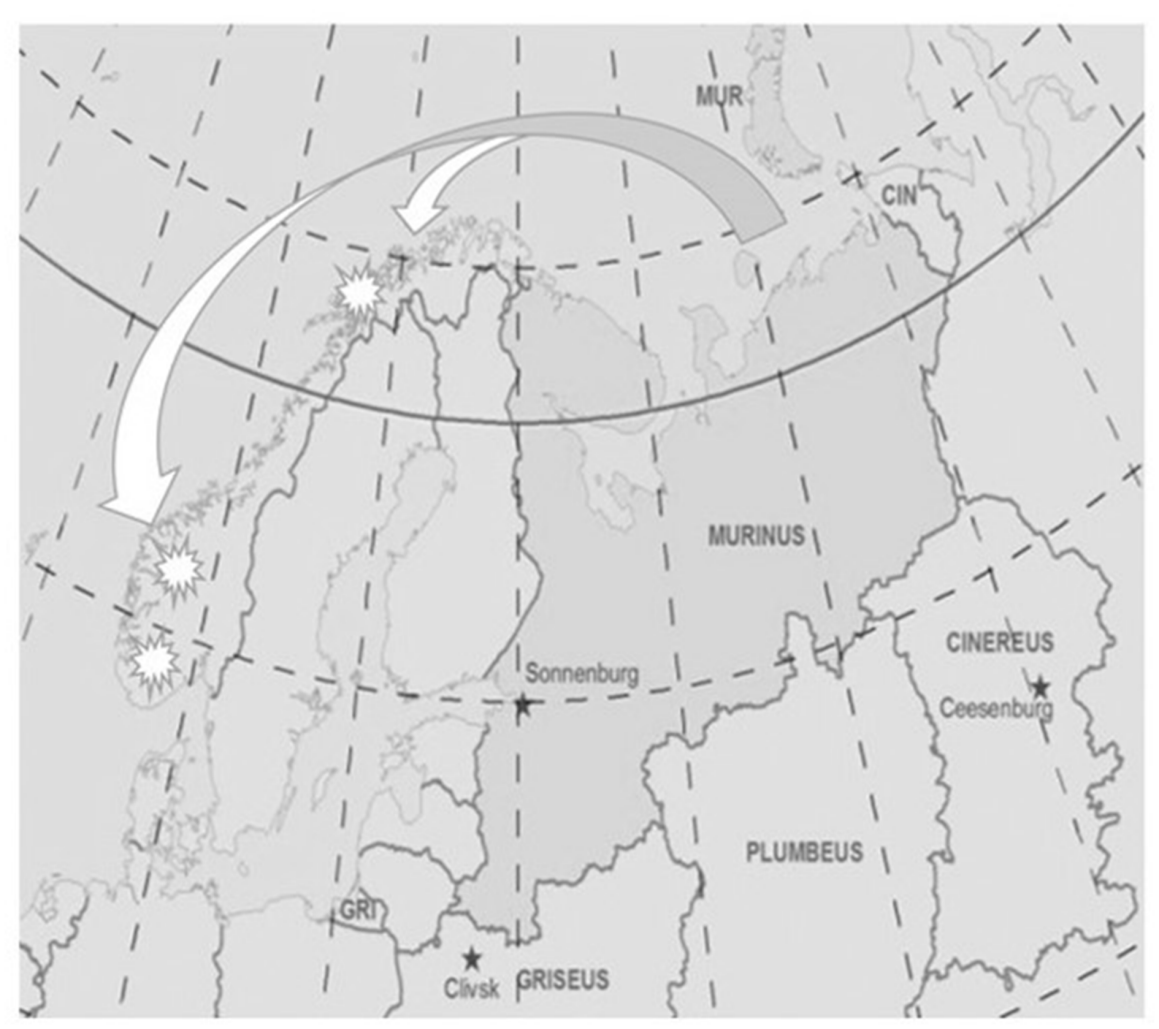
The simulation’s strategic scenario. The scenario was created by the authors from a research planning session. The image and fictitious states of Murinus, Griseus, Plumbeus, and Cinereus are based on the unclassified strategic scenario of NATO’s Occasus exercise module.

To reflect the temporal effects in actual crises, the scenario involved a total of 36 trials, of which each trial concerned hybrid attacks attributable to a hostile state (see Figure 1) that openly opposed the interests of Norway. Each trial was represented by a mission that required action from the participants. All trials involved ambiguity and required different capabilities in areas such as policing, surveillance, close protection, security assistance, high-risk arrests, and direct action. These mission categories represent the types of methods an affected state might utilize when confronting hybrid warfare (Monaghan, 2019).

The first 12 trials represented the peacetime condition, in which a combination of state and non-state adversaries engaged in criminal and irregular activities below the threshold of war. The next 24 trials represented the wartime condition, in which the attacks intensified to include combat actions short of full-scale war, but not sufficiently to invoke Article 5 of the NATO Treaty.

### Exogenous variables

#### Dyad composition

Dyad composition was coded as an ordinal variable based on the implied order of the dyads’ crisis response capabilities (refer to Table 1). As interagency approaches are advocated to counter hybrid warfare (Yanakiev, 2018), we expected that dyads comprising both police and military personnel would demonstrate the highest organizational performance. The mixed police-military dyads were thus ranked in first position. Furthermore, since the police have the leading role in civilian crisis management in Norway (Røksund et al., 2013), while this is a secondary task for the military (Forsvarsdepartementet, 2021), the all-police dyads were ranked second and the all-military dyads were ranked third.

**Table 1:** Ordinal coding of dyad categories.

First	Second	Third
Mixed police-military dyads	All-police dyads	All-military dyads

#### Peacetime performance

Following (Murphy and Cleveland, 1995), performance was measured by two subject matter experts (SMEs) and calculated as a mean score based on the SMEs’ assessment of the dyads’ decision-making regarding task organization and prioritization across the 12 initial trials comprising the simulation’s peace-phase. The SMEs were unaware of participant identities and assigned a score to each dyad per mission on a scale from zero to six (0 = poor and 6 = excellent). The scoring criteria were the dyads’ demonstration of integrated mission management by using the full range of police and military capabilities to change or maintain the efforts necessary to achieve a successful outcome. When the SMEs’ judgments differed, the final score was their average score.

The SMEs were selected on the basis of their prominent levels of academic qualifications and professional knowledge accumulated from more than 30 years of involvement in various security crises. The military SME was a recently retired general with extensive experience from the Norwegian joint headquarters. The police SME was an active-duty chief of police with extensive experience from the Norwegian police directorate. The extent of their competencies thus made them subject matter experts in crisis management.

#### Pre-test collective self-efficacy

Pre-test collective self-efficacy was calculated as a mean score on a 12-item questionnaire. It involved a seven-point Likert scale adapted from an instrument originally developed by Bandura (2006, p. 335). Each item was a measure of generality and required judgments that applied to hybrid warfare issues corresponding in form and difficulty to those in the present scenario. For example, the dyads were asked to rate their beliefs about successfully performing crisis response-related skills in a series of headquarters activities, such as the ability to “efficiently assign tasks to subordinates despite organizational boundaries” and “successfully oversee operations to identify and detain hostile actors.”

A group discussion approach was used, since it reflects the interaction processes between individuals and thus truly represents the group’s beliefs (Gibson et al., 2000). Yet there are concerns that group discussions can turn into a social influence event and be subject to persuasive efforts by influential members to achieve consensus (Stajkovic et al., 2009). In the current study, however, we believe these social concerns were mitigated by our balancing of the participants’ age and seniority (see note 3).^3^

### Endogenous variables

#### Persistence

Following Seo and Ilies (2009), persistence was measured by two elements for each trial: First, the dyads’ instructions regarding “effort expenditure” on a scale from zero to six, as 0 = be very restrictive and 6 = be unrestricted. Second, the dyads’ “resource mobilization” on a scale from zero to four, as 0 = no troops and 4 = more than three troops. A ratio of seven to five between these elements was applied, since the dyads’ effort expenditure was considered to represent persistence to a greater extent than the number of troops allocated to missions. The measure was a sum of the two elements’ mean-scores across the 24 missions comprising the war phase.

#### Wartime performance

The wartime performance variable was calculated by the same SME protocol as the exogenous variable, peacetime performance. As such, wartime performance was a mean score based on the dyads’ task organization and mission prioritization across the 24 trials comprising the war phase.

#### Post-test collective self-efficacy

Succeeding the final trial, post-test collective self-efficacy was collected as a repeated measurement using the same 12-item questionnaire as pre-test collective self-efficacy.

### Procedure

The study’s background information and crisis scenario were emailed to the participants prior to the day of the simulation. The study’s purpose was presented as an assessment of collaborative crisis response, in which the participants’ job was to work together with another participant to manage the operations of a national headquarters. Following Wood et al. (1990), the current study’s decision tasks were developed in line with the theoretical considerations of SCT, and were intended to resemble the actual mission management processes of higher-level headquarters (NATO, 2013). The participants were told they could withdraw at any point, and once the simulation had started, there could be no communication between them and the researcher. All data, including the informed consent form consistent with international ethical standards of scientific research, was collected electronically.

At the start of the simulation, the participants received a scenario update that included strategic guidance and policy instructions. Subsequently, the participants completed the pre-test collective self-efficacy questionnaire. When the questionnaire was completed, the scenario unfolded.

In each trial, the participants’ first decision-task was assigning troops to a given mission. Multiple-choice options were used to organize the available police and military resources into a unit, the participants believed was suitable to accomplish the task at hand. The available police resources consisted of the following: counter-terrorism police, local SWAT teams, police security service, and uniformed armed police. Furthermore, the military resources encompassed special operations forces, home guard rangers, counter-intelligence, and armed military guards. This implies that the participants could choose to mobilize a single resource, up to a maximum of eight resources on a trial.

The second decision-task was to provide intent-instructions regarding the use of force (i.e., how much effort a given set of resources should expend in the face of adversities). The participants expressed their guidance on effort expenditure by positioning a marker on a seven-point Likert scale. The anchor points on the scale were defined as “very restrictive resource expenditure” and “unrestricted resource expenditure.” The midpoint indicated “moderate resource expenditure.” The third decision-task was to prioritize each mission (i.e., high/medium/low) in ways believed to optimize mission execution in temporal terms. The participants expressed their priority guidance by positioning a marker on a seven-point Likert scale. It consisted of anchor points denoting “No priority” and “very high priority.” The midpoint of corresponded to “respond within 24 h.”

The transition from peace to war was established by a royal decree declaring a state of war. This kind of royal decrees are authorized through a special provision in the Norwegian defense act that allows the military to establish police-military cooperation and resist with all means available in the event of an armed attack on Norway (The Constitution of the Kingdom of Norway, 1917, §25). For example, it extends the constraints otherwise imposed on the use of force by peacetime legal processes to include the legitimate killing of enemy combatants and detention of foreign officials until hostilities have subsided (Beredskapsloven, 1950).

Throughout the simulation, participants could choose to reject missions if deemed appropriate. Although resource mobilization and effort expenditure were measured as zero in such cases, reject decisions could produce high performance scores if deemed appropriate by the SMEs. Justifications for rejecting missions were not collected.

### The current study’s path model

To analyze the dyads` organizational performance in wartime, we followed (Bandura, 1997) and applied the individual level model of Wood and Bandura (1989, p. 379) to the collective level in the present analysis. Similarly, the causal pathways in our final model were based on their temporal sequencing in the scenario.

**Figure 2:**
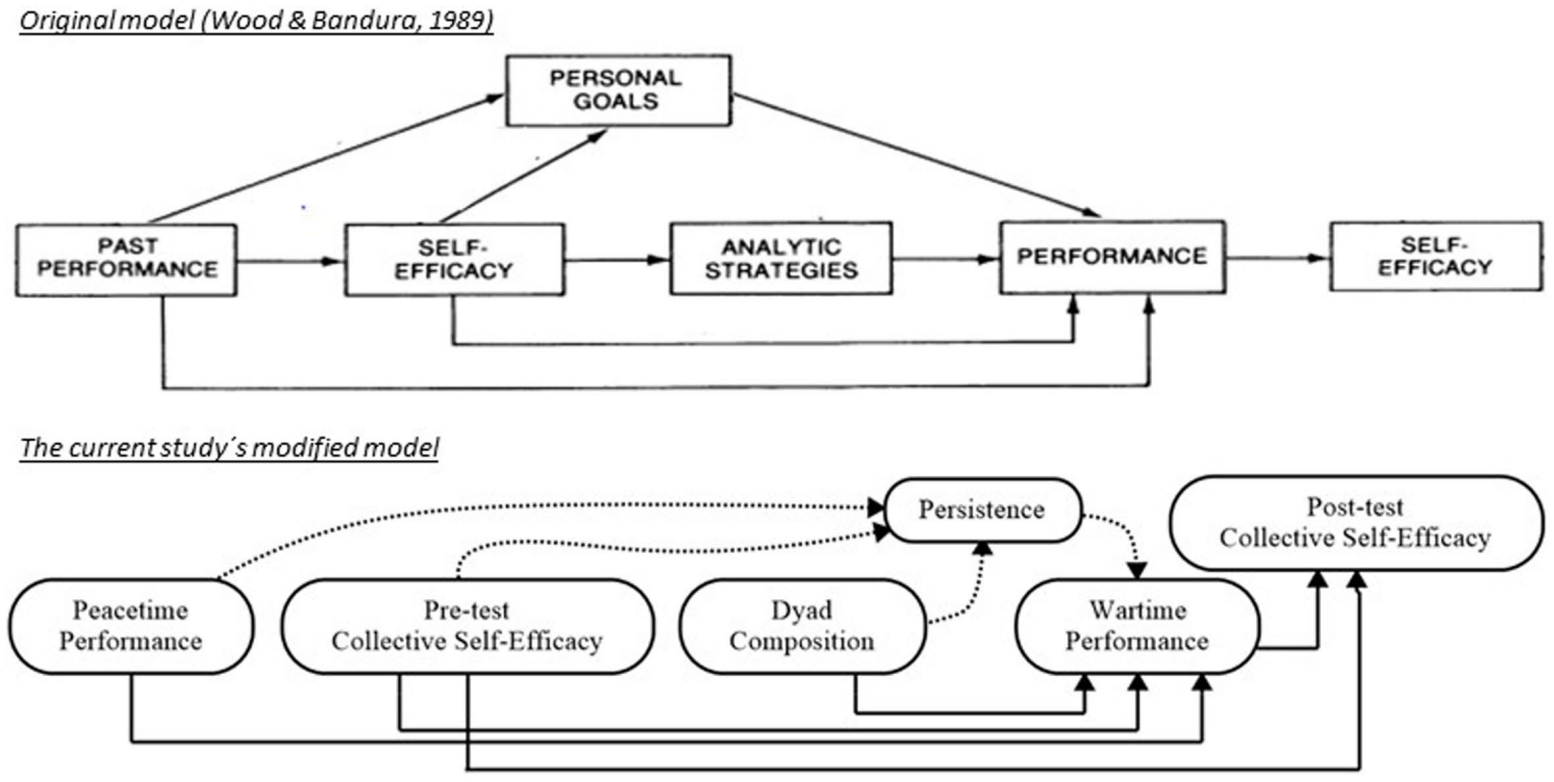
The original model as described by Wood and Bandura (1989) and the present analysis modified model. The original model of Wood and Bandura (1989) comprised two iterations of the one shown in the image.

As illustrated in Figure 2, the following modifications were made to original model of Wood and Bandura (1989): First, the “analytic strategies” endogenous variable was replaced by the “dyad composition” exogenous variable. We believe this was justified, as the participants were assigned to one of three dyad categories, based on their background. In this case, the dyads’ analytical strategies were inherent to the participants’ previous experience (i.e., police or military) and their respective standards of conduct (Bandura and Wood, 1989, p. 810). Secondly, the mediator variable of “personal goals” was replaced by the endogenous variable of “persistence.” Although empirically related, goals and persistence are theoretically distinct constructs that affect performance (Schunk and DiBenedetto, 2021), and we believed persistence would play the most relevant role. Not only because persistence is described as a strong predictor of performance in conditions of high ambiguity (Bandura, 1986), but also because the relationship between goals and performance in ambiguous circumstances is unresolved (Oppi et al., 2022), although reverse relationships have been identified (Jung, 2014). Thirdly, our model included a direct link between pre-test collective self-efficacy and post-test collective self-efficacy. We believe this was justified, as it seems likely that variables other than mere performance feedback would serve as efficacy sources in the current study. On this note, numerous scholars have shown how perceived efficacy predicts future efficacy beliefs (Goddard et al., 2004) and that efficacy beliefs are indeed negatively related to performance in ambiguous circumstances (Schmidt and DeShon, 2010).

### Statistics

The data were analyzed in SPSS Statistics 28.0.1.0. The subsequent path analysis was performed in SPSS AMOS 28.0.0.

For the performance measurements, the SME interrater reliability showed an intraclass correlation of 0.80 (0.76 for peacetime performance and 0.84 for wartime performance), indicating a good reliability among the SMEs. The collective self-efficacy-scale’s Cronbach’s alpha was 0.81 on average (0.78 for pre-test collective self-efficacy and 0.83 for post-test collective self-efficacy). The persistence scale’s Cronbach’s alpha was 0.91 on average (0.87 for “effort expenditure” and 0.95 for “resource mobilization”). All the scales were thus sufficiently consistent to indicate that the measures were reliable. No violations of normality were found. There was no missing data.

## Results

Table 2 presents the means, standard deviations, and Pearson r correlations for the variables. The following two-tailed correlation coefficients were significant at the 0.05 level: (1) Dyad composition with persistence, wartime performance, pre-test collective self-efficacy, and post-test collective self-efficacy; (2) Peace and wartime performance with persistence; (3) Peacetime performance with wartime performance; and (4) Pre-test collective self-efficacy with post-test collective self-efficacy.

**Table 2:** Means (M), standard deviations (SD), and Pearson’s *r* correlations for the variables (*N* = 69).

Correlations								
Variables	1	2	3	4	5	6	M	SD
1. Dyad type	-						-	-
2. Persistence	−0.30^*^	-					6.42	0.93
3. Peacetime performance	0.17	−0.54^**^	-				4.15	0.59
4. Wartime performance	0.27^*^	−0.61^**^	0.66^**^	-			4.57	0.46
5. Pre-test collective self-efficacy	−0.29^*^	−0.03	0.05	0.08	-		3.99	0.61
6. Post-test collective self-efficacy	−0.27^*^	0.02	−0.03	0.04	0.86^**^	-	4.14	0.62

^*^p < 0.05, ^**^p < 0.01, two-tailed (95% confidence level). SD, Standard deviation.

Although the correlation coefficients shed light on all the current study’s hypotheses, a path analysis was necessary to determine the direct and indirect effects of the variables on each other. The final path model yielded a non-significant χ^2^ (3, *N* = 69) of 1.474, *p* = 0.69; a goodness-of-fit index adjusted for degrees of freedom (AGFI) of 0.95; a normed fit index (NFI) of 0.99; and a Tucker–Lewis index (TLI) of 1.05. All indicate an excellent model fit.

**Figure 3:**
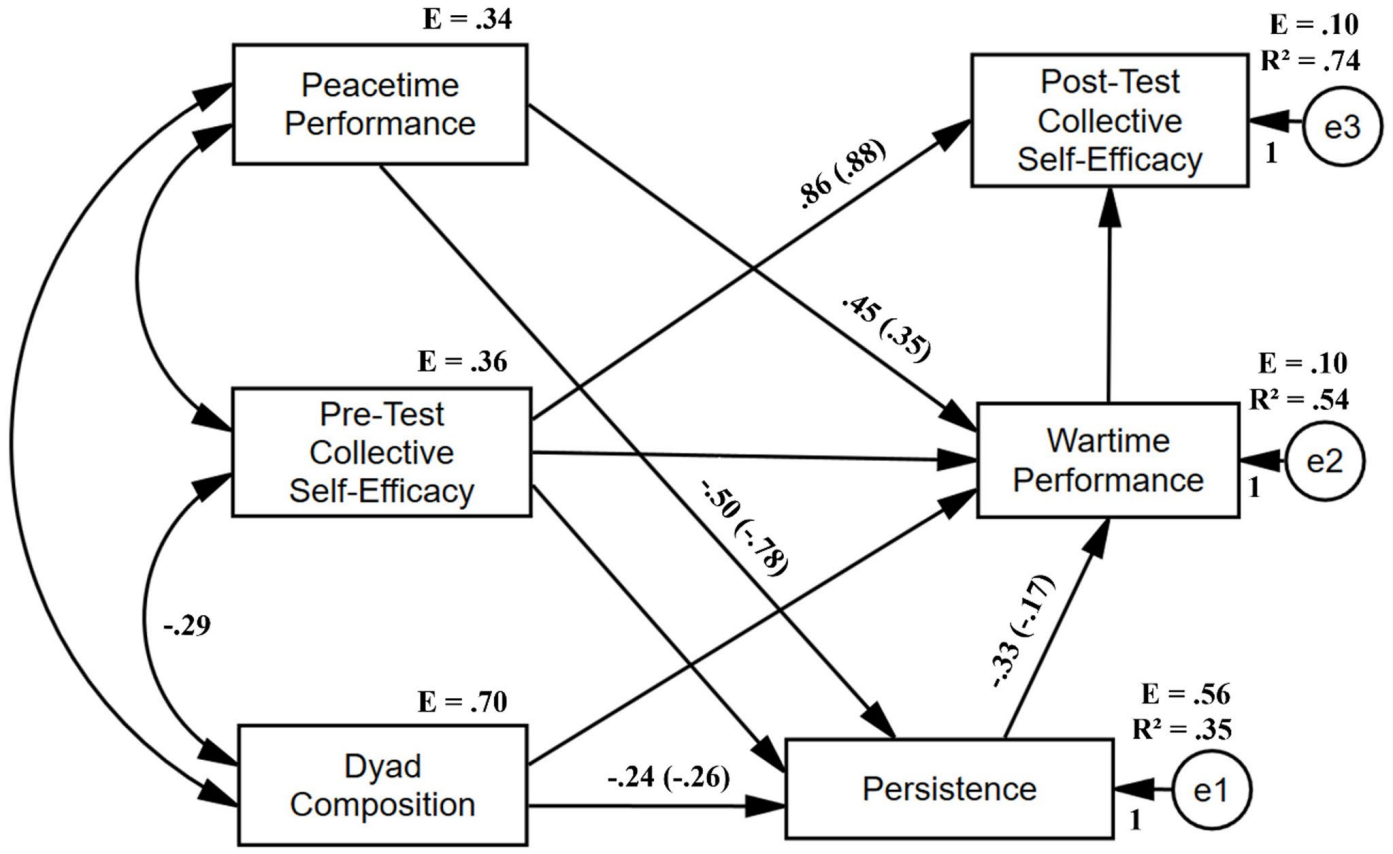
Path analysis output. Path coefficients in the form of standardized regression weights appear outside parentheses. Only statistically significant correlation/path coefficients are shown. Model fit summary: AGFI = 0.950, TLI = 1.045, and NFI = 0.992.

*R*^2^ for wartime performance was 0.54 (*p* < 0.01). *R*^2^ for persistence was 0.35 (*p* < 0.01). *R*^2^ for post-test collective self-efficacy was 0.74 (*p* < 0.01). The path analysis’ outcomes are shown in Table 3 in the form of standardized and unstandardized regression weights. The path model is illustrated in Figure 3.

**Table 3:** Decomposition of effects from path analysis.

Effect	Unstandardized coefficient	SE	Standardized coefficient	Critical ratio	*R*^2^
1. Dyad type	0.06	0.05	0.11	1.20	0.54^**^
2. Peacetime performance	0.35	0.08	0.45	4.59^**^	
3. Persistence	−0.17	0.05	−0.33	−3.27^**^	
4. Pre-test CSE	0.06	0.07	0.08	0.94	
On wartime performance					
1. Dyad type	−0.26	0.12	−0.24	−2.29^*^	0.35^**^
2. Peacetime performance	−0.78	0.16	−0.50	−4.98^**^	
3. Pre-test CSE	−0.11	0.16	−0.07	−0.72	
On persistence					
1. Pre-test CSE	0.88	0.06	0.86	13.98^**^	0.74^**^
2. Wartime performance	−0.05	0.08	−0.03	−0.55	
On post-test CSE					
	Peacetime performance	Pre-test CSE	Wartime performance	Persistence	Dyad composition
Standardized direct effects					
Wartime performance	0.45^**^	0.08	-	−0.33^**^	0.11
Persistence	−0.50^**^	−0.07	-	-	−0.24^*^
Post-test CSE	0.00	0.86^**^	−0.03	-	0.00
Standardized indirect effects					
Wartime performance	0.17^**^	0.03	-	-	0.08^*^
Post-test CSE	−0.02	−0.01	-	0.01	−0.01

^*^p < 0.05, ^**^p < 0.01, two-tailed (95% confidence level). CSE, Collective self-efficacy; SE, Standard error.

The following path coefficients were statistically significant at the 0.05 level: (1) The positive indirect effect of dyad composition on wartime performance through the mediation of persistence, which partially supported hypothesis 1 (H1). (2) The negative direct effect of persistence on performance, which supported hypothesis 4 (H4). (3) The negative direct effect of peacetime performance on persistence. (4) The negative direct effect of dyad composition on persistence. (5) The positive direct effect of peacetime performance on wartime performance, which supported hypothesis 2 (H2). (6) The positive indirect effect of peacetime performance on wartime performance through mediation of persistence, which supported hypothesis 2 (H2). (7) The positive direct effect of pre-test collective efficacy on post-test collective efficacy.

A striking outcome of the present analysis was the non-significant relationship between collective self-efficacy and performance in wartime, which contradicted our hypothesis 3 (H3).

## Discussion

This study illustrates how the organizational performance of police and military commanders working together to counter hybrid warfare involving conventional, irregular, terrorist, and criminal forces was predicted by four social cognitive factors: Dyad composition, past performance in peacetime, collective self-efficacy, and persistence. The latter served as mediator of the others. The result suggests how these factors adequately explained a great deal of the variability in wartime performance. Our analysis thus supports how Bandura (1986) contends that adaptive self-regulation based on people’s standards of adequacy has a strong influence on performance directly, but also indirectly through the mediation of persistence. It also elaborates how Schunk and DiBenedetto (2021) describe the ways social cognitive mechanisms determine how people experiencing discrepancies between standards and perceived progress create selective incentives that can lead to differential outcomes on decisions, persistence, and subsequent performance attainment in ambiguous circumstances. For example, the demonstrated links between dyad composition, persistence, and wartime performance suggest how situational properties activated differential self-evaluations of progress and the usefulness of actions in terms of the dyads’ specific standards.

Our initial core finding clarified our hypothesized benefits of interagency grouping (H1) by indicating a positive indirect effect of dyad composition on wartime performance, mediated by persistence. This means that when dyad composition went up, performance went up too, due to differential effort expenditure and resource mobilization in wartime. Although this finding suggests that the dyads comprised the right competencies, it seems to indicate a degree of difference in their ability to ferret out information and prescribe efficient instructions based on ambiguous feedback. It indicates how the commanders had divergent expectations about the ways in which persistence could produce valued outcomes in the context of hybrid warfare, and has two prominent features. On the one hand, as the mixed dyads were ordered first, followed by the all-police and all-military, it reveals how our result disfavors the former and contradicts H1. On the other hand, as the unmediated path coefficient between dyad composition and wartime performance was non-significant, while the correlation coefficient was significant, it suggests that less persistent dyads achieved the highest wartime performance, which indeed provides support for H4.

Likewise, our second major finding delineates a negative direct effect of persistence on wartime performance. It elaborates the link between reduced persistence and superior performance in wartime by illustrating how more persistent dyads were less efficient than those demonstrating lower persistence. This finding not only provides additional support for H4, but also contradicts social cognitive research explaining that greater persistence leads to higher performance (Schunk, 2012). However, we believe this finding is more in line with descriptions of Bandura (1999) of how ambiguity provides fertile grounds for misjudgment when people seek to discover how outcomes are linked to actions occurring immediately or far removed in time, with different effects depending on where and toward whom they are performed. As admonished by Weick and Sutcliffe (2015, p. 71), such misbeliefs may invoke trade-offs between accuracy and stability, generating interactions that are increasingly removed from what is actually happening. This problem is discussed by Bartone (2010), arguing how security operations that start out with limited aims may quickly escalate to much larger affairs in the direction of misbeliefs. As the aims of crisis response are not only to prevent incidents from escalating into full-scale war, but also to exploit opportunities whenever they occur (Williams, 2021), our interpretation of the negative effect of persistence on performance is that hybrid warfare, like most security crises, requires the ability to exercise control adaptively, based on nuanced understandings of the ambiguous relationship between the perceived appropriateness of tactical actions and strategic aims.

This line of reasoning was elaborated upon by the manner in which our analysis indicated a direct negative effect of peacetime performance on persistence. This result supports how Schunk and DiBenedetto (2021) assert that enhanced past performance predicts reduced persistence, as skilled individuals often have to persist less to succeed in their domain of expertise. On this note, Bandura (1999) describes how enacted mastery experiences strengthen both the skills of decision-makers and their subsequent ease of implementing actions according to standards. In the current study, our result thus suggests how the more efficient dyads in peacetime indeed expected reduced persistence levels to be effective in wartime. We reason that the more efficient dyads embraced ambiguities differently than those less successful. Subsequently, it seems that efficient dyads focused on the dangers of excessive escalation, rather than calling for intense and preemptive countermeasures.

Moreover, the analysis unveiled additional insights regarding dyad composition. While it was observed that the combination of individuals in dyads exerted a favorable indirect influence on wartime performance, it was also observed that dyad composition seemed to be associated with their overall levels of persistence during the simulation. It indicates that when dyad composition went up, persistence went down, which implies that the all-military dyads were least persistent. According to Bandura (1999), the demonstrated differential persistence levels suggest that commanders had contrasting outcome expectations and thus regulated their behavior differently. In this context, escalation appears as a stronger inhibitor of persistence for the all-military dyads, than for the other dyads. Although this is in line with research showing that police commanders prefer more urgent actions than military commanders in war (Mattingsdal et al., 2023), it also contradicts scholars arguing for the military’s inclinations toward the offensive (Posen, 2014). To this end, we follow Bandura (1999) and reason that the police and military’s domain-specific standards played a key role in determining the commanders’ persistence in the face of hybrid warfare.

Additional support for H2 was also demonstrated by the ways the dyad’s past performance in peacetime seemed to have a positive direct effect on actual performance in wartime. It suggests that dyads with enhanced performance in peacetime achieved higher performance in wartime. This supports research describing how past performance is a useful predictor of future performance (Sitzmann and Yeo, 2013). On the contrary, it also emphasizes the cautionary notes put forth by Bandura (1999, p. 171) regarding the potential negative consequences of overreliance on past accomplishments on tasks that require deliberate and thoughtful action. In the context of warfare, for instance, if commanders become complacent and fail to adjust to change based on their earlier successes, this could ultimately result in a deterioration of their overall performance. Consequently, this underscores the importance for commanders to engage in regular situational assessments and critically evaluate the relevance of their past accomplishments. By doing so, commanders can better determine whether the strategies employed in the past remain applicable within the present context.

In light of this observation, our analysis offered further support for H2 by demonstrating how wartime performance was influenced positively by peacetime performance through the mediation of persistence. By considering the mediating role of persistence, we can understand that it is not merely the dyad’s past performance itself that directly influences their wartime performance but rather their effort expenditure and resource mobilization fostered through their actions in more permissive circumstances. This lesson indicates how the successful organizing of resources in peacetime promoted a purposeful transfer of judgments beyond the initial peacetime condition. It also raises the question of whether those involved in crisis response (i.e., task organizing) are better predictors of wartime performance than how persistent they are.

However, as both our indirect findings favors the latter, our analysis suggests that commanders engaged in decision-making regarding hybrid warfare are more likely to determine their levels of persistence by evaluating environmental factors more than by comparing their present and past achievements, from one perspective. Whereas this interpretation is less convincing from the perspective of the demonstrated direct effect of peacetime performance. Thus, one could argue that these two perspectives are not in direct contradiction, they simply focus on different aspects of triadic reciprocality and reflect the asymmetric influences among the environment, person, and behavior in collaborative crisis response. Ultimately, within the context of hybrid warfare, this underscores the importance of commanders maintaining a high degree of adaptability to change in order to uphold optimal persistence levels, which, in turn, could serve as a foundation for achieving success in this ambiguous environment.

With regard to our assertions on collective self-efficacy, our findings yielded more varied outcomes compared to the results obtained for the aforementioned factors. Although the commanders’ efficacy beliefs remained consistent from the beginning to the end of the simulation, the relationship between collective self-efficacy and their overall performance levels during the simulation did not appear to be significant. Similarly, efficacy beliefs did not seem to exert a substantial influence on persistence, which contradicts research showing that strong efficacy beliefs are a reliable predictor of greater persistence (Schunk and DiBenedetto, 2021). However, as pre-test collective self-efficacy explained a great deal of the variability in post-test collective self-efficacy, our result supports how Bandura (1997) and recent research (Malmberg et al., 2014) describe repeated mastery experiences as potent sources of efficacy. Even though we did not directly measure mastery experience, it indicates how most dyads interpreted their efforts favorably and lends some empirical evidence to elaborate on how professionals develop efficacy beliefs for interdependent tasks over time in ambiguous circumstances.

As such, our analysis did not provide support for the theorized link between collective self-efficacy and performance (H3). This contradicts previous studies indicating that higher collective self-efficacy is related to higher performance (Stajkovic et al., 2009), but supports how Bandura (1997) contends that even the strongest efficacy beliefs will not lead to performance attainments, unless the environment in which groups function provides appropriate opportunities for success. Thus, this could be interpreted as indicating that the simulation’s ambiguous feedback did not provide room for efficient self-directed forethought. Bandura (1999) describes how such ambiguous tasks may require more emphasis on external consequences than on efficacy beliefs, to exercise control of one’s actions, and how domain-specific expertise is required to achieve high levels of performance in such contexts. Hence, our results highlight the importance of police and military commanders engaging in a collective and deliberate consideration of the external outcomes that are deemed relevant. This practice has the potential to generate valuable information that complements their individual domain-specific expertise, especially in the context of cross-sectoral endeavors. Moreover these insights could enable them to make more strategic decisions, even when faced with ambiguity that may instill doubts about their ability to master a given activity.

Consequently, our analysis suggest how the efficacy beliefs of actors affected by hybrid warfare are likely to play a weak role in predicting their persistence and subsequent performance. We reason that these findings underscore why scholars argue that there is a need for prudent decision-making in times of war (Gray, 2010). For example, the simulation involved translating strategic goals into feasible actions that called for broad direction and long-term alignment of functions based on misinformation and hardly any evidence of impact. Hybrid warfare seemingly differs from research in less ambiguous settings showing great effects of collective self-efficacy on performance in work such as education (Donohoo et al., 2018), sports (Eccles and Tenenbaum, 2007), and healthcare (Smith et al., 2018).

A limitation of the present study is that path analysis only provides suggestions for ways that the processes examined influence each other (Sobel, 2000). On this basis, more definitive causal relationships could be followed up through research to analyze the links between behavioral, situational, and personal factors of decision makers. For example, how persistence predicts performance in triads or larger groups. We also recommend repeated measures research that compares between group differences in performance using police and military participants in scenarios involving collaborative responses to man-made or natural disasters.

A second limitation is the use of persistence as an endogenous variable. Although “effort expenditure” and “resource mobilization” are well-established in SCT (Bandura, 1986, p. 394) as indicators of persistent intent, it is important to note that the scale producing this variable was developed without a large-scale validation study. Nonetheless, analysis establishing standardized scores that measure persistence more accurately in collaborative crisis response is an important direction for future research.

## Conclusion

In this analysis, we illustrated several important links between dyad composition, past performance in peacetime, collective self-efficacy, and persistence among police and military commanders working together in times of war. The results support research explaining the influence of adaptive self-regulation in ambiguous circumstances. It also highlights the arguments of scholars asserting that cross-sectoral dialogue is needed for the police and military commanders to prepare themselves for the challenges of the contemporary security environment, whether it involves public safety or national security. In this context, our core finding is how the standards selected as a mark of adequacy are essential for guiding interagency efforts to the successful achievement of strategic progress. We believe these results are critical for promoting police-military interoperability in the management of hybrid warfare and other transboundary threats. To this end, three implications emerge from our analysis.

Firstly, if it is true that lower persistence leads to higher performance attainment and that the influence of collective self-efficacy is weak, then commanders would be well-advised to assess their use of force-instructions throughout the conduct of a collaborative crisis response. Similarly, commanders should pay more attention to people’s domain-specific skills than their perceived ability to accomplish tasks, as such perceptions are less likely to accurately predict their actual performance in wartime. Secondly, if the lack of skills leads to resource waste and risks of excessive escalation, then more realistic training exercises would be useful to improve performance by reducing transaction costs in decision-making to the extent that they increase the police and military’s capacity to counter hybrid warfare. Finally, if it is true that peacetime performance predicts performance in war, then our results provide empirical evidence that the Norwegian security frameworks developed in peacetime are indeed efficient when responding to hybrid attacks in times of war.

## Data availability statement

The raw data supporting the conclusions of this article will be made available by the authors, without undue reservation.

## Ethics statement

The studies involving humans were approved by Norwegian Agency for Shared Services in Education and Research. The studies were conducted in accordance with the local legislation and institutional requirements. The participants provided their written informed consent to participate in this study. Written informed consent was obtained from the individual(s) for the publication of any potentially identifiable images or data included in this article.

## Author contributions

JM, JA, BJ, and RE: conceptualization and writing—review and editing. JM and BJ: methodology. JM and JA: investigation. JM: formal analysis, funding acquisition, project administration, and writing—original draft. JM, BJ, and RE: resources. RE and BJ supervised JM. All authors contributed to the article and approved the submitted version.

## Footnotes

^1^In the report of Cullen and Reichborn-Kjennerud (2017), hybrid warfare is defined as the synchronized use of multiple instruments of power tailored to specific vulnerabilities across the full spectrum of societal functions, to achieve synergistic effects. The report asserts that hybrid warfare is a widespread approach that is likely to grow as a security challenge, justifying new efforts by nations to understand the threat it presents.

^2^The term “Western countries” refers to nations that are typically located in North America, Western Europe, and other regions where Western cultural, political, and economic influences are prevalent. These countries often share similar democratic systems, values, and institutions (McNeill, 1997). While the specific list of countries considered “Western” may vary depending on the context, examples generally include the United States, United Kingdom, Germany, Norway, and other countries within Western Europe and North America.

^3^To test the balancing of the participants’ age and seniority, an ANOVA test was conducted. It showed no significant effects of age or seniority for any of the dyads.
